# Occupational Disparities in Cancer Survival Among the Working Population in Japan: 10‐Year Survival Analysis Using the Kanagawa Cancer Registry

**DOI:** 10.1002/cam4.71020

**Published:** 2025-07-03

**Authors:** Kazuhiko Watanabe, Ichiro Kawachi, Masayoshi Zaitsu

**Affiliations:** ^1^ Center for Research of the Aging Workforce, University of Occupational and Environmental Health, Japan Kitakyushu Fukuoka Japan; ^2^ Department of Social and Behavioral Sciences Harvard T.H. Chan School of Public Health Boston Massachusetts USA

**Keywords:** cancer, disparity, occupational class, survival

## Abstract

**Background:**

Limited data exist on occupational disparities in long‐term cancer mortality among the working‐age population in Japan. We examined occupational disparities in long‐term cancer survival, focusing on 10‐year survival outcomes among working‐age populations.

**Methods:**

This retrospective observational study used data from the Kanagawa Cancer Registry of 41,632 patients with cancer aged 20–65 years who were diagnosed between 1992 and 2015, with a 10‐year follow‐up. Patients were classified into four occupational classes based on their longest‐held occupations (upper nonmanual, lower nonmanual, manual, and primary industry). The primary outcome was all‐cause mortality, and cancer‐specific mortality was the secondary outcome. Poisson regression was used to estimate the mortality rate ratios (MRRs) and 95% confidence intervals (CIs) for each occupational class, adjusted for sex, age, and year of diagnosis. Additional analyses were performed for common cancer sites (stomach, lung, colorectal, and breast).

**Results:**

MRRs for all‐cause mortality were higher in lower nonmanual (MRR = 1.14, 95% CI 1.10–1.18), manual (MRR = 1.38, 95% CI 1.32–1.43), and primary industry workers (MRR = 1.19, 95% CI 1.09–1.31) than in upper nonmanual workers (professional and managerial occupations). Similar patterns were observed across common cancer sites and cancer‐specific mortality. Adjusting for cancer stage and treatment attenuated these disparities but did not eliminate them, particularly among manual workers.

**Conclusions:**

We observed occupational disparities in long‐term cancer mortality among working‐age populations in Japan, with manual workers experiencing worse survival outcomes. Promoting targeted interventions, healthy lifestyles, and early cancer detection for cancer survivors in the workplace are crucial for mitigating these disparities.

## 1 Introduction

Cancer remains a global public health concern, with 20 million new cases and nearly 10 million deaths reported in 2022 [1]. In Japan, cancer is the leading cause of death, accounting for approximately 385,000 deaths (223,000 men and 162,000 women) in 2020 [2]. Despite advancements in early detection and treatment, the cancer burden continues to increase.

Japan faces an urgent challenge of ensuring the long‐term health of its aging workforce. The 2021 revision of the Act on Stabilization of Employment of Elderly Persons mandates companies to secure employment until workers are aged 65 years and encourages efforts to extend employment opportunities up to 70 years [3]. Although the system of universal health coverage in Japan provides equitable access to standardized cancer treatment, emerging evidence documents the existence of socioeconomic disparities in cancer mortality, particularly across occupational classes [4, 5, 6, 7, 8]. These disparities pose significant concerns for the aging workforce in Japan, with profound implications for public health and economic stability.

Currently, data specifically addressing occupational disparities in cancer prognosis among actively employed populations are limited. In a previous study, individuals whose longest‐held occupations were in manual occupational classes (e.g., manufacturing, construction, and transportation) had lower 5‐year cancer prognoses than those in upper nonmanual occupational classes (e.g., professional and managerial roles) [8]. Approximately one‐third of the excess mortality risk was attributed to advanced cancer stage at diagnosis [8]. However, this study did not specifically focus on actively employed populations, as many participants had already retired at baseline. As retirement policies have evolved in Japan, evaluating cancer prognosis over extended periods—such as 10‐year survival rates—instead of the conventional 5‐year rates, may be crucial for ensuring the long‐term health of the aging workforce [9].

In this study, we aimed to examine occupational disparities in long‐term cancer survival, focusing on 10‐year survival outcomes among working‐age populations. Additionally, we sought to identify mortality disparities by cancer type, emphasizing common cancers for which workplace screening programs are well‐established in Japan.

## 2 Methods

### 2.1 Study Design and Study Participants

This longitudinal study used data from the Kanagawa Cancer Registry (KCR) to conduct a 10‐year survival analysis of patients with incident cancer aged 20–65 years diagnosed between 1992 and 2015. The details of the KCR have been described previously [10, 11, 12]. Briefly, the KCR is one of the largest population‐based cancer registries in Japan, covering a catchment of approximately 9 million people, that gathers general (sex, age, date of cancer diagnosis, date of death, or last follow‐up) and clinical information (cancer site, stage, and treatment). Additionally, occupational information of patients at the time of cancer diagnosis (based on their longest‐held occupation) was collected until 2015 [8, 11, 12]. The occupational distribution in the KCR is nationally representative [11, 12].

From a total of 851,547 patients with cancer registered in the KCR between 1992 and 2015 with a first‐ever cancer diagnosis (C00–C97 and D06 in the International Classification of Diseases, 10th revision; Table 1) and no duplicate cancer cases, the following patients were excluded: (a) those aged < 20 or > 65 years (*n* = 514,115); (b) those with missing occupational information (*n* = 276,643); (c) homemakers, students, and unemployed individuals (*n* = 18,206); and (d) those engaged in security occupations (e.g., self‐defense forces personnel, police officers, Japan Coast Guard officers, and firefighters) (*n* = 951). These exclusions resulted in a final dataset of 41,632 patients with cancer having complete occupational information for analysis.

**TABLE 1:** All‐cause and cancer‐specific 10‐year survival estimates in overall and common cancer sites.

Cancer site	ICD‐10	Participants, *n*	Age, mean (SD), years	Women, %	10‐year survival estimate, % (95% CI)^a^	
					All‐cause	Cancer‐specific
All sites	C00–97, D06	41,632	54 (8)	33.7	46.3 (45.8–46.8)	52.4 (51.9–52.9)
Specific site						
Stomach	C16	8465	55 (8)	23.7	50.2 (49.1–51.2)	57.0 (56.0–58.1)
Lung	C33, 34	5752	56 (7)	22.1	18.9 (17.9–19.9)	21.9 (20.9–23.1)
Colon and rectum	C18–20	7733	55 (8)	29.4	56.9 (55.8–58.0)	64.1 (63.0–65.1)
Breast	C50	6697	49 (8)	100.0	78.2 (77.2–79.2)	80.0 (79.0–80.9)

Abbreviations: CI, confidence interval; ICD‐10, International Classification of Diseases, 10th revision; SD, standard deviation.

^a^ Survival estimates were calculated using the Kaplan–Meier method.

A de‐identified dataset was obtained under a research agreement between the authors and the KCR. The ethics committee of the University of Occupational and Environmental Health, Japan, approved the study protocol (number: R4‐054).

### 2.2 Main Outcome

The primary outcome was all‐cause mortality, with person‐years calculated from the date of the initial cancer diagnosis to the date of death or last follow‐up. Cancer‐specific mortality was the secondary outcome. For specific cancer sites, we focused on five major common sites—the stomach, lung, colon, rectum, and breast—for which well‐established cancer screening systems are available in Japan (Table 1 and Table S1). In the cancer‐specific mortality analysis, observations were censored if the cause of death was a cancer site other than the initial diagnosis [8].

### 2.3 Occupational Class and Other Variables

In accordance with prior studies using the longest‐held occupation data from the KCR [8, 11, 12], individuals were classified into four occupational groups: upper nonmanual workers (professional and managerial workers), lower nonmanual workers (clerical, sales, and service workers), manual workers (manufacturing, construction, mining, and transportation workers), and primary industry workers (Table S1) [8]. Covariates included basic confounding factors, such as sex, age, and year of diagnosis. We included advanced stage (Union for International Cancer Control Tumor–Node–Metastasis Stages III–IV) and receipt of any cancer treatment (surgery, chemotherapy, or radiation therapy) as prognostic variables to explain occupational disparities in cancer survival [8]. In Japan, cancer care is provided under a universal health insurance system, and treatments are generally administered in accordance with standardized clinical guidelines. Therefore, we considered this classification (surgery, chemotherapy, or radiation therapy) to reasonably capture the major therapeutic strategies and serve as an appropriate adjustment for a potential mediator, despite possible variations in specific regimens or treatment intensity.

### 2.4 Statistical Analysis

The 10‐year all‐cause and cancer‐specific survival rates were estimated using the Kaplan–Meier method. In our primary analytical model (Model 1), Poisson regression was used to estimate the mortality rate ratios (MRRs) and 95% confidence intervals (CIs) for all‐cause and cancer‐specific mortalities, adjusted for basic confounding variables (sex, age, and year of diagnosis). Upper nonmanual workers served as the reference group for all analyses. We further adjusted for two prognostic variables (cancer stage and treatment) to examine how these variables could explain the observed occupational disparities (Model 2). However, this additional analysis was limited to patients with complete information regarding cancer stage and treatment (*n* = 3543). Overdispersion was calculated by dividing the deviance by the degree of freedom (deviance/df) in each model. We also estimated MRRs using Poisson regression with robust variance to assess the robustness of the results. Subgroup analyses applied the same methods to specific cancer sites (the stomach, lung, colon, rectum, and breast). For sensitivity analyses, MRRs were estimated and stratified by age (20–49, 50–59, and 60–65 years).

## 3 Results

During the 10‐year period (median follow‐up time, 10 years), the all‐cause and cancer‐specific survival rates of all the participants were 46.3% and 52.4%, respectively (Table 1). Manual workers had significantly lower survival rates, with 36.4% for all‐cause survival and 43.5% for cancer‐specific survival, than those of upper nonmanual workers (professional and managerial occupations), whose survival rates were 51.5% and 56.9%, respectively (Table 2). These differences were consistent across all common cancer sites, with advanced cancer stages being most prevalent among manual workers (Table 2).

**TABLE 2:** Background characteristics and outcomes by occupational class.

Characteristics	Occupational class^a^			
	Upper nonmanual	Lower nonmanual	Manual	Primary industry
Overall				
Basic characteristics, *n*	8541	19,848	12,342	901
Women, *n* (%)	**2945 (35%)**	**9642 (49%)**	**1257 (10%)**	**199 (22%)**
Age, mean (SD), years	**54 (9)**	**54 (8)**	**56 (8)**	**58 (7)**
Year of diagnosis, mean (SD)	**1998 (5)**	**1998 (4)**	**1997 (4)**	**1997 (4)**
10‐year OS, %	**51.5**	**50.6**	**36.4**	**39.0**
10‐year CSS, %	**56.9**	**56.2**	**43.5**	**45.3**
Prognostic variable, *n* ^b^	922	1621	957	43
Advanced stage, *n* (%)	**579 (63%)**	**1017 (63%)**	**710 (74%)**	**26 (61%)**
Any treatment, *n* (%)	**902 (98%)**	**1572 (97%)**	**906 (95%)**	**43 (100%)**
Stomach				
Basic characteristics, *n*	1623	3735	2887	220
Women, *n* (%)	**386 (24%)**	**1417 (38%)**	**167 (6%)**	**34 (16%)**
Age, mean (SD), years	**55 (8)**	**54 (8)**	**56 (7)**	**59 (6)**
Year of diagnosis, mean (SD)	**1997 (4)**	**1997 (4)**	**1997 (4)**	**1997 (4)**
10‐year OS, %	**54.7**	**52.4**	**45.2**	**44.1**
10‐year CSS, %	**60.4**	**58.6**	**53.5**	**50.3**
Prognostic variable, *n* ^b^	138	275	157	12
Advanced stage, *n* (%)	**81 (59%)**	**141 (51%)**	**108 (69%)**	**6 (50%)**
Any treatment, *n* (%)	137 (99%)	274 (99%)	152 (97%)	12 (100%)
Lung				
Basic characteristics, *n*	1000	2430	2187	135
Women, *n* (%)	**231 (23%)**	**887 (37%)**	**142 (7%)**	**11 (8%)**
Age, mean (SD), years	**55 (9)**	**56 (8)**	**57 (7)**	**59 (6)**
Year of diagnosis, mean (SD)	**1998 (5)**	**1998 (4)**	**1998 (4)**	**1997 (4)**
10‐year OS, %	**21.9**	**21.0**	**15.8**	**9.6**
10‐year CSS, %	**25.6**	**23.7**	**18.7**	**14.8**
Prognostic variable, *n* ^b^	147	307	298	9
Advanced stage, *n* (%)	111 (76%)	229 (75%)	244 (82%)	8 (89%)
Any treatment, *n* (%)	143 (97%)	284 (93%)	276 (93%)	9 (100%)
Colon and rectum				
Basic characteristics, *n*	1719	3705	2150	159
Women, *n* (%)	**455 (27%)**	**1572 (42%)**	**190 (9%)**	**54 (34%)**
Age, mean (SD), years	**54 (8)**	**55 (7)**	**56 (7)**	**58 (8)**
Year of diagnosis, mean (SD)	**1998 (5)**	**1998 (4)**	**1998 (4)**	**1997 (4)**
10‐year OS, %	**58.9**	**58.6**	**52.5**	**54.7**
10‐year CSS, %	**65.4**	**65.5**	**60.7**	**60.4**
Prognostic variable, *n* ^b^	181	331	222	9
Advanced stage, *n* (%)	130 (72%)	248 (75%)	166 (75%)	7 (78%)
Any treatment, *n* (%)	180 (99%)	327 (99%)	216 (97%)	9 (100%)
Breast				
Basic characteristics, *n*	1501	4546	575	75
Women, *n* (%)	1501 (100%)	4546 (100%)	575 (100%)	75 (100%)
Age, mean (SD), years	**48 (9)**	**49 (8)**	**51 (7)**	**54 (8)**
Year of diagnosis, mean (SD)	**1999 (5)**	**1997 (4)**	**1998 (4)**	**1998 (3)**
10‐year OS, %	**80.4**	**77.8**	**74.8**	**81.3**
10‐year CSS, %	**82.3**	**79.7**	**76.3**	**81.3**
Prognostic variable, *n* ^b^	232	399	44	4
Advanced stage, *n* (%)	112 (48%)	198 (50%)	21 (48%)	2 (50%)
Any treatment, *n* (%)	232 (100%)	398 (99.7%)	44 (100%)	4 (100%)

*Note:* Bold faces indicate *p* < 0.05 for analysis of variance, Chi‐squared test, or log‐rank test.

Abbreviations: CSS, cancer‐specific survival; OS, overall survival; SD, standard deviation.

^a^ Upper nonmanual workers include professional and managerial workers; lower nonmanual workers include clerical, sales, and service workers; manual workers include manufacturing, construction, mining, and transportation workers; primary industry workers include agriculture, forestry, and fishery workers.

^b^ Advanced stages included tumor–node–metastasis Stages III–IV; any treatment included surgery, chemotherapy, or radiation therapy.

**FIGURE 1:**
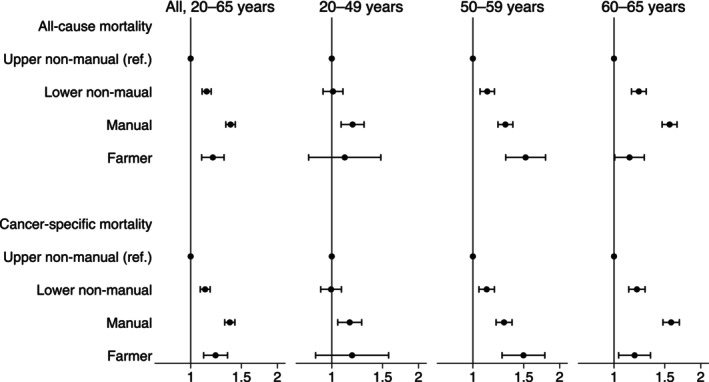
All‐cause and cancer‐specific mortality rate ratios across different occupational classes estimated with Poisson regression. Mortality rate ratios (dots) and 95% confidence intervals (bars) for overall and cancer‐specific deaths were estimated using a Poisson regression adjusted for age and year of diagnosis. The number of patients analyzed was as follows: All patients aged 20–65 years, *n* = 41,632; patients aged 20–49 years, *n* = 10,296; patients aged 50–59 years, *n* = 17,507; and patients aged 60–65 years, *n* = 13,829.

In regression analyses, lower nonmanual workers (MRR = 1.14, 95% CI 1.10–1.18), manual workers (MRR = 1.38, 95% CI 1.32–1.43), and primary industry workers (MRR = 1.19, 95% CI 1.09–1.31) had significantly higher all‐cause mortality than those of upper nonmanual workers (Figure 1 and Table 3). Similar patterns were observed in cancer‐specific mortality. In Model 2, after adjusting for cancer stage and treatment, the observed disparities were attenuated but remained significant for manual workers in all‐cause mortality (MRR = 1.34) and cancer‐specific mortality (MRR = 1.37, Table 3). Model fit was assessed using deviance divided by the degree of freedom. The deviance/df values were above 1 in the basic model (model 1), suggesting overdispersion (e.g., 2.54 for stomach cancer in all‐cause mortality). In the fully adjusted model (model 2), the values were closer to 1 (ranging from 0.78 to 1.84), indicating improved model fit and reduced overdispersion (Table S1). In sensitivity analyses using Poisson regression with robust variance, the associations between the occupational group and mortality remained consistent. Manual workers showed significantly higher all‐cause and cancer‐specific mortalities compared to those in the upper nonmanual worker group across common cancer sites (Table S2).

**TABLE 3:** Mortality rate ratios and 95% confidence intervals for all‐cause and cancer‐specific mortalities estimated with Poisson regression.

Longest‐held occupation	Mortality rate ratio (95% confidence interval)							
	10‐year all‐cause mortality				10‐year cancer‐specific mortality			
	Death, *n*	Model 1^a^	Death, *n*	Model 2^b^	Death, *n*	Model 1^a^	Death, *n*	Model 2^b^
Overall		*n* = 41,632		*n* = 3543		*n* = 41,632		*n* = 3543
Upper nonmanual	4145	Reference	395	Reference	3603	Reference	330	Reference
Lower nonmanual	9813	**1.14 (1.10–1.18)**	684	1.02 (0.90–1.16)	8495	**1.12 (1.08–1.17)**	577	1.03 (0.90–1.18)
Manual	7844	**1.38 (1.32–1.43)**	571	**1.34 (1.17–1.53)**	6741	**1.37 (1.31–1.42)**	487	**1.37 (1.19–1.58)**
Primary industry	550	**1.19 (1.09–1.31)**	23	1.45 (0.95–2.22)	481	**1.22 (1.11–1.34)**	20	**1.57 (1.00–2.48)**
Stomach		*n* = 8465		*n* = 582		*n* = 8465		*n* = 582
Upper nonmanual	735	Reference	62	Reference	635	Reference	48	Reference
Lower nonmanual	1779	**1.10 (1.01–1.20)**	107	1.12 (0.81–1.54)	1516	1.07 (0.98–1.18)	88	1.25 (0.87–1.80)
Manual	1583	**1.33 (1.22–1.45)**	74	1.12 (0.79–1.60)	1308	**1.29 (1.17–1.42)**	65	1.38 (0.93–2.05)
Primary industry	123	**1.33 (1.10–1.61)**	4	0.98 (0.35–2.70)	108	**1.41 (1.15–1.73)**	4	1.47 (0.53–4.12)
Lung		*n* = 5752		*n* = 761		*n* = 5752		*n* = 761
Upper nonmanual	781	Reference	102	Reference	726	Reference	90	Reference
Lower nonmanual	1921	**1.13 (1.04–1.23)**	211	1.01 (0.80–1.29)	1813	**1.15 (1.06–1.26)**	193	1.05 (0.81–1.35)
Manual	1842	**1.21 (1.11–1.32)**	230	1.05 (0.83–1.34)	1720	**1.22 (1.12–1.33)**	210	1.10 (0.85–1.42)
Primary industry	122	**1.24 (1.03–1.51)**	8	1.14 (0.55–2.35)	110	1.22 (0.99–1.49)	7	1.13 (0.52–2.45)
Colon and rectum		*n* = 7733		*n* = 743		*n* = 7733		*n* = 743
Upper nonmanual	706	Reference	67	Reference	582	Reference	57	Reference
Lower nonmanual	1534	1.05 (0.96–1.15)	130	1.02 (0.76–1.37)	1247	1.03 (0.93–1.14)	103	0.95 (0.68–1.31)
Manual	1021	**1.16 (1.06–1.28)**	109	1.36 (0.99–1.87)	818	**1.15 (1.03–1.28)**	77	1.11 (0.78–1.58)
Primary industry	72	1.04 (0.81–1.32)	8	**3.11 (1.49–6.52)**	62	1.10 (0.85–1.43)	6	**2.63 (1.13–6.14)**
Breast (female)		*n* = 6697		*n* = 679		*n* = 6697		*n* = 679
Upper nonmanual	294	Reference	44	Reference	264	Reference	39	Reference
Lower nonmanual	1008	1.09 (0.96–1.24)	76	0.89 (0.61–1.30)	918	1.11 (0.97–1.27)	68	0.90 (0.61–1.35)
Manual	145	**1.24 (1.02–1.52)**	12	1.32 (0.70–2.51)	136	**1.32 (1.07–1.62)**	12	1.51 (0.79–2.89)
Primary industry	14	0.86 (0.50–1.48)	0	—	14	1.00 (0.58–1.71)	0	—

*Note:* Boldface indicates *p* < 0.05.

^a^ Adjusted for basic confounding factors (age, sex, and year of diagnosis).

^b^ Additional adjustments for prognostic variables (cancer stage and treatment).

In subgroup analyses, manual workers had significantly lower all‐cause and cancer‐specific survival across all common cancer sites, including colorectal cancers, compared to their nonmanual counterparts (Table 3). Occupational disparities were pronounced in the older working population (i.e., > 50 years) in the sensitivity analyses (Figure 1 and Table S3).

## 4 Discussion

This study identified significant occupational disparities in 10‐year all‐cause and cancer‐specific mortality rates among working‐age Japanese populations. Manual workers exhibited poorer prognoses for all‐cause and cancer‐specific survival, with a mortality risk approximately 1.4 times higher than that of nonmanual workers. Notably, these occupational disparities were consistently observed across all the common cancer sites.

Our findings are partially consistent with those of previous studies [8, 11, 13]. To our best knowledge, this study is the first to comprehensively investigate occupational disparities in long‐term cancer prognosis among working‐age populations. Previous research has highlighted disparities in lung, stomach, and breast cancers [8], and this study further extends these findings to include worse colorectal cancer survival among manual workers. These results suggest that occupational disparities may be prevalent across all common cancers included in workplace cancer screening programs in Japan despite universal health coverage designed to ensure equitable access to cancer care. The differences in participation in cancer screening across occupations may partly explain the observed prognostic disparities [14]. In Japan, while workplace screening programs for major common cancers, such as stomach, lung, colorectal, and breast cancers, are well established, participation rates remain suboptimal and vary across socioeconomic backgrounds [15, 16, 17]. In addition to household income and education level, factors, such as the ease of taking leave, availability of time for cancer screening, workplace culture, awareness of cancer screening, and overall health consciousness, may influence cancer screening behaviors [15, 16, 17, 18]. Additionally, larger company sizes might be associated with higher cancer screening participation rates [16]. Therefore, these aforementioned factors should be carefully considered to better understand and address occupational disparities in cancer screening participation. These disparities in participation may directly influence the cancer stage at diagnosis [19]. Approximately one‐third of occupational disparities in excess mortality risk can be attributed to differences in advanced cancer stages [8]. Consistent with these findings, our study observed a significant attenuation of disparities after adjusting for the cancer stage, indicating that an advanced stage at diagnosis plays a key role in these disparities. Moreover, prognostic disparities were more pronounced in older age groups (aged ≥ 50 years) than in younger age groups, which represent the primary target population of workplace cancer screening programs.

In addition to clinical factors such as cancer stage and treatment, patients' lifestyle behaviors—including diet, smoking cessation, alcohol abstinence, regular exercise, and adequate sleep—may contribute to improved life expectancy and long‐term prognosis [20]. Maintaining healthy lifestyle behaviors, such as engaging in regular physical activity while continuing to work, could help reduce the risk of cancer recurrence [21, 22] and contribute to improved long‐term outcomes for patients with cancer. Thus, the observed prognostic disparities might be partly explained by differences in adherence to a healthy lifestyle.

Beyond screening participation, structural barriers might also contribute to occupational disparities in cancer outcomes. Manual workers often face physical demands, extended working hours, and limited flexibility in scheduling medical appointments, including screening and treatment [19, 23]. These obstacles may delay access to timely medical interventions and exacerbate the disparities identified in this study [24]. Additionally, psychosocial stress associated with physically demanding jobs may be linked to delayed help‐seeking behavior [25], potentially resulting in later‐stage diagnoses and poorer outcomes in disadvantaged groups [24].

Addressing these disparities requires systematic changes to workplace policies. Workplace health initiatives should specifically address the needs of individuals who continue to work after being diagnosed with and treated for cancer in the workforce. With increasing life expectancy, the number of employed individuals with cancer has been increasing, with an estimated 499,000 cancer outpatients remaining in the workforce—half of whom are aged ≥ 60 years [26]. Tailored interventions are essential to reduce barriers to care for manual workers among young to older aged workers. Flexible scheduling of medical appointments, onsite health education programs, promotion of healthy lifestyle habits, and employer‐provided incentives to encourage screening participation can help mitigate these challenges. Additionally, employers should foster a supportive environment by implementing leave policies, raising workplace awareness, encouraging colleague support, and addressing psychological stress related to treatment to improve workforce participation and well‐being. Integrating workplace health initiatives with national healthcare policies could ensure equitable access to cancer care and sustaining long‐term employment for affected workers.

This study has some limitations. First, although our dataset was derived from a population‐based cancer registry, missing occupational data limited its generalizability. The excluded group with missing occupational data had a higher proportion of women compared with the included group with available data. This may have introduced selection bias. Specifically, if women with better prognosis were more likely to be excluded, the mortality risk may have been underestimated in our analysis (Table S4). Individuals with missing occupation data showed significantly lower survival, which may reflect unmeasured sociodemographic or clinical factors. In addition, the occupation recorded in the registry may not necessarily reflect the occupation at the exact time of cancer diagnosis, and we were unable to determine how many individuals changed their occupation afterward. However, the occupational distribution in the KCR is regarded as nationally representative [11, 12]. Second, we were unable to assess the influence of other relevant socioeconomic indicators, such as educational attainment and income, or lifestyle factors, including smoking, alcohol consumption, and physical activity, which could mediate the observed associations [20, 21, 22, 27]. Further research is needed to confirm whether differences in adherence to healthy lifestyle behaviors cause occupational disparities in long‐term mortality. Third, the use of broad categories for cancer staging and treatment may have resulted in misclassifications. Finally, although the fully adjusted model (Model 2) showed substantially improved goodness‐of‐fit with reduced deviance/df values across outcomes and cancer sites, the number of outcome events was limited in some occupational groups. Among workers in the primary industry sector, the small number of events may have reduced the statistical power of subgroup analyses in Model 2. Therefore, results from these specific subgroups should be interpreted with caution. In addition, owing to the limited number of cervical cancer cases, we were unable to assess disparities in this cancer type, which is currently included in workplace cancer screening programs. In Japan, the participation rate in uterine/cervical cancer screening over a 2‐year period was 43.6% among individuals aged 20–69 years [28], and it was even lower among workers at 34.3% [29]. Among the common cancer sites, improving screening participation remains a critical public health challenge.

Despite these limitations, this study presents novel evidence of significant occupational disparities in long‐term cancer prognosis among the working‐age Japanese population. As employment beyond the age of 65 years becomes increasingly common, addressing occupational disparities in cancer prognosis—a leading cause of death—remains a pressing social issue. Prioritizing tailored workplace interventions for cancer survivors in the workplace and integrating them with broader public health initiatives are crucial for achieving equity in cancer outcomes.

## 5 Conclusions

This study confirmed occupational disparities in long‐term cancer mortality among working‐age populations, with manual workers experiencing poorer outcomes than their nonmanual counterparts. These findings underscore the urgent need to address occupational health disparities through targeted workplace interventions for cancer survivors, promoting early cancer detection and ensuring equitable access to care to support the well‐being and productivity of a diverse and aging workforce.

## Author Contributions

**Kazuhiko Watanabe:** conceptualization (equal), data curation (supporting), formal analysis (supporting), investigation (supporting), methodology (equal), validation (equal), visualization (equal), writing – original draft (lead), writing – review and editing (equal). **Ichiro Kawachi:** conceptualization (equal), formal analysis (supporting), methodology (equal), project administration (equal), resources (supporting), writing – original draft (supporting), writing – review and editing (equal). **Masayoshi Zaitsu:** conceptualization (lead), data curation (lead), formal analysis (lead), funding acquisition (lead), investigation (lead), methodology (equal), project administration (lead), resources (lead), supervision (lead), validation (equal), visualization (equal), writing – original draft (supporting), writing – review and editing (equal).

## Ethics Statement

A de‐identified dataset was obtained under a research agreement between the authors and the Kanagawa Cancer Registry, and the ethics committee of the University of Occupational and Environmental Health, Japan, approved the study protocol (number: R4‐054).

## Consent

The need for informed consent was waived because the data set of the Kanagawa Cancer Registry is collected in accordance with Japanese national law and provided for use as secondary data, as it is anonymously processed information.

## Conflicts of Interest

The authors declare no conflicts of interest.

## Supporting information


**Table S1.** Evaluation of model fit using deviance divided by degrees of freedom (deviance/df) from Poisson regression models without robust variance.
**Table S2**. Mortality rate ratios and 95% confidence intervals for all‐cause and cancer‐specific mortalities estimated with Poisson regression with robust variance.
**Table S3**. Results of Poisson regression for 10‐year overall and cancer‐specific mortalities stratified by age.
**Table S4**. Background characteristics and outcomes by availability of occupational information.

## Data Availability

The data that support the findings of this study are available from the corresponding author upon reasonable request. Requests to access the original database can be made by following the procedures described in the URL: http://www.pref.kanagawa.jp/docs/nf5/ganntaisaku/know‐about‐gan/ganntouroku‐deta‐start.html.
