# Supplementary material for: Occupational Disparities in Cancer Survival Among the Working Population in Japan: 10‐Year Survival Analysis Using the Kanagawa Cancer Registry
Source: Cancer Med. 2025 Jul 3;14(13):e71020. doi: 10.1002/cam4.71020 (PMC12223785; doi:10.1002/cam4.71020)
Supplement: Supplementary file 1 — Table S1. Evaluation of model fit using deviance divided by degrees of freedom (deviance/df) from Poisson regression models without robust variance. Table S2. Mortality rate ratios and 95% confidence intervals for all‐cause and cancer‐specific mortalities estimated with Poisson regression with robust variance. Table S3. Results of Poisson regression for 10‐year overall and cancer‐specific mortalities stratified by age. Table S4. Background characteristics and outcomes by availability of occupational information. [file CAM4-14-e71020-s001.docx]

**Supporting Information**

**Occupational disparities in cancer survival among the working population in Japan: 10-year survival analysis using the Kanagawa Cancer Registry**

Kazuhiko Watanabe, RPT, PhD^1^; Ichiro Kawachi, MD, PhD^2^; Masayoshi Zaitsu, MD, PhD^1^

^1^Center for Research of the Aging Workforce, University of Occupational and Environmental Health, Japan, Kitakyushu, Fukuoka, Japan

^2^Department of Social and Behavioral Sciences, Harvard T.H. Chan School of Public Health, Boston, Massachusetts, USA

**Table S1. Evaluation of model fit using deviance divided by degrees of freedom (deviance/df) from Poisson regression models without robust variance**

|  | Outcome | | | |
| --- | --- | --- | --- | --- |
|  | All-cause mortality | | Cancer-specific mortality | |
| Cancer site | Model 1 | Model 2 | Model 1 | Model 2 |
| Overall | 2.41 | 1.67 | 2.36 | 1.57 |
| Stomach | 2.54 | 1.58 | 2.48 | 1.41 |
| Lung | 2.49 | 1.84 | 2.50 | 1.81 |
| Colorectal | 1.97 | 1.44 | 1.86 | 1.32 |
| Breast | 1.12 | 0.83 | 1.08 | 0.78 |

Values present the model fit statistics (deviance/ df) for Poisson regression models prior to applying robust variance. Deviance/df values closer to 1 suggest better goodness-of-fit.

**Table S2. Mortality rate ratios and 95% confidence intervals for all-cause and cancer-specific mortalities estimated with Poisson regression with robust variance**

| Longest-held occupation | Mortality rate ratio (95% confidence interval) | | | | | | | | |
| --- | --- | --- | --- | --- | --- | --- | --- | --- | --- |
|  | 10-year all-cause mortality | | | |  | 10-year cancer-specific mortality | | | |
|  | Death, n | Model 1^†^ | Death, n | Model 2^‡^ |  | Death, n | Model 1^†^ | Death, n | Model 2^‡^ |
| *Overall* |  | n=41,632 |  | n=3,543 |  |  | n=41,632 |  | n=3,543 |
| Upper non-manual | 4,145 | Reference | 395 | Reference |  | 3,603 | Reference | 330 | Reference |
| Lower non-manual | 9,813 | **1.14 (1.09–1.19)** | 684 | 1.02 (0.88–1.19) |  | 8,495 | **1.12 (1.07–1.18)** | 577 | 1.03 (0.88–1.21) |
| Manual | 7,844 | **1.38 (1.31–1.44)** | 571 | **1.34 (1.13–1.58)** |  | 6,741 | **1.37 (1.30–1.44)** | 487 | **1.37 (1.15–1.64)** |
| Primary industry | 550 | **1.19 (1.07–1.33)** | 23 | 1.45 (0.89–2.37) |  | 481 | **1.22 (1.08–1.38)** | 20 | 1.57 (0.91–2.71) |
| *Stomach* |  | n=8,465 |  | n=582 |  |  | n=8,465 |  | n=582 |
| Upper non-manual | 735 | Reference | 62 | Reference |  | 635 | Reference | 48 | Reference |
| Lower non-manual | 1,779 | 1.10 (0.995–1.22)^*^ | 107 | 1.12 (0.75–1.66) |  | 1,516 | 1.07 (0.96–1.20) | 88 | 1.25 (0.78–1.98) |
| Manual | 1,583 | **1.33 (1.20–1.48)** | 74 | 1.12 (0.72–1.75) |  | 1,308 | **1.29 (1.15–1.45)** | 65 | 1.38 (0.83–2.30) |
| Primary industry | 123 | **1.33 (1.04–1.69)** | 4 | 0.98 (0.29–3.30) |  | 108 | **1.41 (1.08–1.82)** | 4 | 1.47 (0.39–5.53) |
| *Lung* |  | n=5,752 |  | n=761 |  |  | n=5,752 |  | n=761 |
| Upper non-manual | 781 | Reference | 102 | Reference |  | 726 | Reference | 90 | Reference |
| Lower non-manual | 1,921 | **1.13 (1.00–1.28)** | 211 | 1.01 (0.74–1.39) |  | 1,813 | **1.15 (1.01–1.31)** | 193 | 1.05 (0.75–1.47) |
| Manual | 1,842 | **1.21 (1.07–1.37)** | 230 | 1.05 (0.76–1.45) |  | 1,720 | **1.22 (1.07–1.39)** | 210 | 1.10 (0.78–1.55) |
| Primary industry | 122 | 1.24 (0.94–1.65) | 8 | 1.14 (0.41–3.19) |  | 110 | 1.22 (0.90–1.65) | 7 | 1.13 (0.39–3.29) |
| *Colon & rectum* |  | n=7,733 |  | n=743 |  |  | n=7,733 |  | n=743 |
| Upper non-manual | 706 | Reference | 67 | Reference |  | 582 | Reference | 57 | Reference |
| Lower non-manual | 1,534 | 1.05 (0.95–1.16) | 130 | 1.02 (0.74–1.41) |  | 1,247 | 1.03 (0.92–1.15) | 103 | 0.95 (0.67–1.34) |
| Manual | 1,021 | **1.16 (1.05–1.30)** | 109 | 1.36 (0.96–1.93) |  | 818 | **1.15 (1.02–1.29)** | 77 | 1.11 (0.75–1.63) |
| Primary industry | 72 | 1.04 (0.80–1.34) | 8 | **3.11 (1.42–6.84)** |  | 62 | 1.10 (0.83–1.46) | 6 | 2.63 (0.97–7.10) |
| *Breast (female)* |  | n=6,697 |  | n=679 |  |  | n=6,697 |  | n=679 |
| Upper non-manual | 294 | Reference | 44 | Reference |  | 264 | Reference | 39 | Reference |
| Lower non-manual | 1,008 | 1.09 (0.95–1.24) | 76 | 0.89 (0.62–1.29) |  | 918 | 1.11 (0.96–1.28) | 68 | 0.90 (0.61–1.34) |
| Manual | 145 | **1.24 (1.01–1.53)** | 12 | 1.32 (0.70–2.49) |  | 136 | **1.32 (1.06–1.63)** | 12 | 1.51 (0.79–2.88) |
| Primary industry | 14 | 0.86 (0.49–1.53) | 0 | – |  | 14 | 1.00 (0.56–1.76) | 0 | – |

† Adjusted for basic confounding factors (age, sex, and year of diagnosis).

‡ Additional adjustments for prognostic variables (cancer stage and treatment).

* Value is shown to 3 decimal places to improve interpretability near statistical thresholds.

Boldface indicates P<0.05.

**Table S3. Results of Poisson regression for 10-year overall and cancer-specific mortalities stratified by age**

| Longest-held occupation | Mortality rate ratio (95% confidence interval) | | | | | | | |
| --- | --- | --- | --- | --- | --- | --- | --- | --- |
|  | 10-year overall mortality | | | | 10-year cancer-specific mortality | | | |
|  | Death | Model 1^†^ | Death | Model 2^‡^ | Death | Model 1^†^ | Death | Model 2^‡^ |
| 20–49 years | | | | | | | | |
| *Overall* |  | n=10,296 |  | n=720 |  | n=10,296 |  | n=720 |
| Upper non-manual | 883 | Reference | 70 | Reference | 821 | Reference | 62 | Reference |
| Lower non-manual | 1,980 | 1.01 (0.93–1.09) | 128 | 0.98 (0.73–1.32) | 1821 | 0.99 (0.92–1.08) | 109 | 0.94 (0.68–1.29) |
| Manual | 1,083 | **1.18 (1.08–1.29)** | 55 | 1.13 (0.78–1.65) | 977 | **1.15 (1.05–1.27)** | 47 | 1.04 (0.70–1.56) |
| Primary industry | 49 | 1.11 (0.83–1.48) | 2 | 1.56 (0.38–6.40) | 48 | 1.18 (0.88–1.57) | 2 | 1.78 (0.43–7.36) |
| *Stomach* |  | n=1,895 |  | n=87 |  | n=1,895 |  | n=87 |
| Upper non-manual | 175 | Reference | 9 | Reference | 167 | Reference | 7 | Reference |
| Lower non-manual | 381 | 0.91 (0.76–1.09) | 20 | 0.61 (0.27–1.40) | 368 | 0.92 (0.76–1.10) | 19 | 0.71 (0.29–1.76) |
| Manual | 233 | 1.00 (0.82–1.23) | 9 | 1.40 (0.52–3.83) | 212 | 0.96 (0.78–1.19) | 9 | 1.74 (0.60–5.05) |
| Primary industry | 11 | 1.51 (0.82–2.78) | 0 | NA | 10 | 1.46 (0.77–2.76) | 0 | NA |
| *Lung* |  | n=1,002 |  | n=95 |  | n=1,002 |  | n=95 |
| Upper non-manual | 164 | Reference | 13 | Reference | 161 | Reference | 13 | Reference |
| Lower non-manual | 344 | 1.08 (0.90–1.30) | 30 | 1.21 (0.61–2.43) | 335 | 1.07 (0.89–1.30) | 25 | 0.88 (0.43–1.83) |
| Manual worker | 245 | 1.11 (0.90–1.36) | 22 | 0.70 (0.32–1.53) | 236 | 1.09 (0.89–1.35) | 21 | 0.55 (0.25–1.20) |
| Primary industry | 9 | **2.70 (1.37–5.32)** | 1 | **9.07 (1.15–71.7)** | 9 | **2.78 (1.41–5.49)** | 1 | **10.3 (1.29–82.3)** |
| *Colon & rectum* |  | n=1,545 |  | n=128 |  | n=1,545 |  | n=128 |
| Upper non-manual | 155 | Reference | 17 | Reference | 138 | Reference | 13 | Reference |
| Lower non-manual | 298 | 1.10 (0.90–1.34) | 28 | 0.93 (0.50–1.72) | 255 | 1.06 (0.86–1.31) | 21 | 0.95 (0.47–1.92) |
| Manual worker | 113 | 0.99 (0.78–1.27) | 8 | 0.99 (0.42–2.35) | 100 | 0.98 (0.76–1.28) | 5 | 0.80 (0.28–2.27) |
| Primary industry | 8 | 0.81 (0.40–1.64) | 0 | NA | 8 | 0.90 (0.44–1.83) | 0 | NA |
| *Breast* |  | n=3,451 |  | n=298 |  | n=3,451 |  | n=298 |
| Upper non-manual | 152 | Reference | 20 | Reference | 145 | Reference | 20 | Reference |
| Lower non-manual | 434 | 1.08 (0.90–1.30) | 24 | 0.64 (0.35–1.16) | 416 | 1.09 (0.90–1.32) | 23 | 0.61 (0.33–1.12) |
| Manual worker | 57 | **1.50 (1.10–2.03)** | 4 | 1.28 (0.43–3.76) | 56 | **1.55 (1.14–2.12)** | 4 | 1.27 (0.43–3.74) |
| Primary industry | 7 | 1.87 (0.88–4.00) | 0 | NA | 7 | 1.97 (0.92–4.22) | 0 | NA |
| 50–59 years | | | | | | | | |
| *Overall* |  | n=17,507 |  | n=1,510 |  | n=17,507 |  | n=1,510 |
| Upper non-manual | 1,636 | Reference | 172 | Reference | 1431 | Reference | 145 | Reference |
| Lower non-manual | 4,255 | **1.12 (1.06–1.19)** | 275 | 0.97 (0.80–1.18) | 3730 | **1.12 (1.05–1.19)** | 231 | 0.98 (0.79–1.20) |
| Manual | 3,388 | **1.30 (1.22–1.38)** | 255 | 1.22 (0.995–1.50) | 2933 | **1.28 (1.20–1.37)** | 225 | **1.29 (1.03–1.61)** |
| Primary industry | 168 | **1.52 (1.30–1.79)** | 7 | 0.94 (0.44–2.02) | 145 | **1.50 (1.26–1.78)** | 6 | 0.94 (0.41–2.15) |
| *Stomach* |  | n=3,563 |  | n=277 |  | n=3,563 |  | n=277 |
| Upper non-manual | 285 | Reference | 32 | Reference | 238 | Reference | 26 | Reference |
| Lower non-manual | 723 | 1.13 (0.98–1.30) | 46 | 0.88 (0.55–1.40) | 606 | 1.11 (0.96–1.30) | 35 | 0.93 (0.55–1.59) |
| Manual worker | 664 | **1.35 (1.18–1.56)** | 26 | 0.64 (0.37–1.11) | 556 | **1.37 (1.17–1.60)** | 21 | 0.72 (0.39–1.35) |
| Primary industry | 36 | **1.65 (1.16–2.33)** | 2 | 0.62 (0.14–2.66) | 30 | **1.67 (1.14–2.44)** | 2 | 0.83 (0.19–3.65) |
| *Lung* |  | n=2,415 |  | n=310 |  | n=2,415 |  | n=310 |
| Upper non-manual | 296 | Reference | 43 | Reference | 279 | Reference | 40 | Reference |
| Lower non-manual | 820 | 1.14 (0.996–1.30) | 78 | 1.46 (0.998–2.12) | 770 | 1.13 (0.98–1.30) | 72 | 1.44 (0.97–2.13) |
| Manual | 767 | 1.13 (0.99–1.29) | 103 | 1.28 (0.87–1.87) | 728 | 1.14 (0.99–1.31) | 98 | 1.30 (0.87–1.93) |
| Primary industry | 28 | 1.46 (0.99–2.14) | 1 | 0.32 (0.04–2.35) | 26 | 1.43 (0.96–2.14) | 1 | 0.34 (0.05–2.53) |
| *Colon & rectum* |  | n=3,610 |  | n=341 |  | n=3,610 |  | n=341 |
| Upper non-manual | 290 | Reference | 29 | Reference | 247 | Reference | 24 | Reference |
| Lower non-manual | 718 | 1.06 (0.92–1.22) | 59 | 0.90 (0.58–1.41) | 617 | 1.06 (0.91–1.23) | 47 | 0.87 (0.53–1.42) |
| Manual | 485 | 1.12 (0.97–1.30) | 52 | 1.18 (0.73–1.89) | 388 | 1.06 (0.91–1.25) | 40 | 1.08 (0.64–1.83) |
| Primary industry | 15 | 0.88 (0.52–1.47) | 3 | 2.32 (0.69–7.82) | 14 | 0.94 (0.55–1.62) | 2 | 1.98 (0.45–8.58) |
| *Breast* |  | n=2,466 |  | n=261 |  | n=2,466 |  | n=261 |
| Upper non-manual | 95 | Reference | 18 | Reference | 78 | Reference | 13 | Reference |
| Lower non-manual | 428 | 1.11 (0.89–1.39) | 34 | 1.10 (0.60–2.00) | 381 | 1.19 (0.93–1.53) | 29 | 1.37 (0.69–2.72) |
| Manual worker | 70 | 1.26 (0.92–1.72) | 6 | 1.40 (0.54–3.62) | 63 | 1.37 (0.98–1.91) | 6 | 1.90 (0.70–5.15) |
| Primary industry | 6 | 0.92 (0.40–2.11) | 0 | NA | 6 | 1.12 (0.49–2.59) | 0 | NA |
| 60–65 years | | | | | | | | |
| *Overall* |  | n=13,829 |  | n=1,313 |  | n=13,829 |  | n=1,313 |
| Upper non-manual | 1,626 | Reference | 153 | Reference | 1351 | Reference | 123 | Reference |
| Lower non-manual | 3,578 | **1.22 (1.15–1.29)** | 281 | 1.13 (0.93–1.38) | 2944 | **1.20 (1.13–1.28)** | 237 | 1.17 (0.94–1.45) |
| Manual | 3,373 | **1.56 (1.47–1.65)** | 261 | **1.50 (1.22–1.83)** | 2831 | **1.58 (1.48–1.68)** | 215 | **1.53 (1.22–1.92)** |
| Primary industry | 333 | **1.13 (1.01–1.27)** | 14 | **2.12 (1.22–3.69)** | 288 | **1.18 (1.04–1.34)** | 12 | **2.64 (1.45–4.81)** |
| *Stomach* |  | n=3,007 |  | n=218 |  | n=3,007 |  | n=218 |
| Upper non-manual | 275 | Reference | 21 | Reference | 230 | Reference | 15 | Reference |
| Lower non-manual | 675 | **1.21 (1.05–1.40)** | 41 | **1.75 (1.02–3.00)** | 542 | 1.15 (0.98–1.34) | 34 | **2.21 (1.19–4.12)** |
| Manual worker | 686 | **1.52 (1.32–1.75)** | 39 | 1.69 (0.97–2.95) | 540 | **1.44 (1.23–1.68)** | 35 | **2.17 (1.15–4.08)** |
| Primary industry | 76 | 1.23 (0.96–1.59) | 2 | 2.59 (0.58–11.5) | 68 | **1.33 (1.01–1.74)** | 2 | **6.77 (1.48–30.9)** |
| *Lung* |  | n=2,335 |  | n=356 |  | n=2,335 |  | n=356 |
| Upper non-manual | 321 | Reference | 46 | Reference | 286 | Reference | 37 | Reference |
| Lower non-manual | 757 | **1.20 (1.05–1.37)** | 103 | 0.74 (0.51–1.05) | 708 | **1.25 (1.09–1.44)** | 96 | 0.81 (0.55–1.19) |
| Manual | 830 | **1.39 (1.22–1.58)** | 105 | 0.73 (0.51–1.05) | 756 | **1.42 (1.24–1.63)** | 91 | 0.76 (0.51–1.13) |
| Primary industry | 85 | 1.15 (0.90–1.46) | 6 | 1.56 (0.67–3.67) | 75 | 1.14 (0.88–1.47) | 5 | 1.61 (0.63–4.11) |
| *Colon & rectum* |  | n=2,578 |  | n=274 |  | n=2,578 |  | n=274 |
| Upper non-manual | 261 | Reference | 21 | Reference | 197 | Reference | 20 | Reference |
| Lower non-manual | 518 | 1.03 (0.88–1.19) | 43 | 1.30 (0.77–2.21) | 375 | 0.98 (0.83–1.17) | 35 | 1.10 (0.63–1.91) |
| Manual | 423 | **1.31 (1.12–1.53)** | 49 | **1.73 (1.02–2.93)** | 330 | **1.35 (1.13–1.61)** | 32 | 1.20 (0.67–2.13) |
| Primary industry | 49 | 1.11 (0.82–1.51) | 5 | **4.07 (1.50–11.0)** | 40 | 1.20 (0.86–1.69) | 4 | **3.34 (1.12–9.99)** |
| *Breast* |  | n=780 |  | n=120 |  | n=780 |  | n=120 |
| Upper non-manual | 47 | Reference | 6 | Reference | 41 | Reference | 6 | Reference |
| Lower non-manual | 146 | 1.11 (0.80–1.54) | 18 | 1.24 (0.49–3.14) | 121 | 1.05 (0.74–1.50) | 16 | 1.09 (0.43–2.81) |
| Manual worker | 18 | 0.83 (0.48–1.44) | 2 | 1.81 (0.35–9.32) | 17 | 0.91 (0.52–1.61) | 2 | 2.08 (0.40–10.9) |
| Primary industry | 1 | 0.15 (0.02–1.10) | 0 | NA | 1 | 0.17 (0.02–1.27) | 0 | NA |

† Adjusted for basic confounding factors (age and year of diagnosis).

‡ Further adjusted for prognostic variables (cancer stage and treatment).

Boldface indicates P<0.05 for Poisson regression.

Abbreviation: NA, not applicable.

**Table S4. Background characteristics and outcomes by availability of occupational information**

| Characteristics | Occupational information | |
| --- | --- | --- |
|  | Available | Missing^*^ |
| Basic characteristics, n | 60,789 | 276,643 |
| Women, n (%) | **16,999 (28%)** | **122,274 (44%)** |
| Age, mean (SD), years | **54 (8)** | **56 (8)** |
| Year of diagnosis, mean (SD) | **1998 (4)** | **2003 (7)** |
| 10-year OS, % | **47.2** | **47.1** |
| 10-year CSS, % | **53.2** | **52.8** |
| Diagnosis stage, n | 5,817 | 95,648 |
| Advanced stage, n^†^ (%) | **3,697 (64%)** | **47,813 (50%)** |
| Treatment, n | 60,321 | 234,224 |
| Any treatment, n^‡^ (%) | **52,467 (87%)** | **201,303 (86%)** |

^*^ Individuals with missing occupation information excluded in this study.

^†^ Advanced stages included tumor–node–metastasis staging Ⅲ–Ⅳ.

^‡^ Any treatment included surgery, chemotherapy, or radiation therapy.

Boldface indicates P<0.05 for the t-test, chi-squared test, or log-rank test.

Abbreviations: OS, overall survival; CSS, cancer-specific survival; SD, standard deviation.
